# CRISPR-Cas13 and polyphenol nanotherapy: a dual-action frontier against renal fibrosis

**DOI:** 10.1097/MS9.0000000000004528

**Published:** 2025-12-09

**Authors:** Khadija Rameen, Manahil Shoukat, Muhammad Talha, Mahnoor Fatima, Ubaid Ur Rehman

**Affiliations:** a Department of Medicine, IBADAT International University, Islamabad, Pakistan; b Department of Medicine, King Edward Medical University, Lahore, Pakistan; c Department of Medicine, Shaheed Ziaur Rahman Medical College, Rajshahi Medical University, Bogura, Bangladesh

*To the Editor,*

Chronic kidney disease (CKD) primarily develops from renal fibrosis, a disease now also visible in individuals displaying symptoms resembling metabolic syndrome. Research and polls have shown that factors including metabolic changes, loss of polyphenols, and intestinal imbalance exacerbate inflammation and kidney scarring^[1]^. These results point to an evolving connection between nutrition, gut flora, and fibrotic kidney disorders, therefore creating opportunities for fresh treatment options. Regrettably, current drugs have shown somewhat limited ability to stop the development of illnesses. Recent data show that urinary polyphenol metabolites might be good biomarkers for diagnosis and therapy monitoring^[2]^. This is in line with the TITAN Guidelines on the need for transparency in AI use in healthcare^[3]^.

This helps to explain how polyphenols might help to lower fibrosis, inflammation, and oxidative stress. Less investigated, the eggplant’s calyx is high in polyphenols and might offer a bright new medicinal source. Using RNA-silencing approaches, nearly a 55% reversal of fibrosis markers was reached in laboratory experiments on kidney organoids utilizing these polyphenols. Already proven in animal models^[4]^, the CRISPR-Cas13a system has the benefit of selectively silencing damaging long non-coding RNAs without permanently changing DNA. Using Cas13a supplied via lipid nanoparticles with orally consumed nano-formulated eggplant polyphenols, a dual-therapy strategy might improve general treatment results. This mix combines a realistic nanomedicine plan with the possibility of greatly enhancing CKD treatment.

The use of Cas13-mediated RNA silencing with natural polyphenols proves to be an innovation for treating renal fibrosis, especially in patients affected by metabolic syndrome. Cas13 is a flexible RNA-targeting tool that can silence harmful transcripts with accuracy, making it a practical option for future therapies^[5]^. In nephrology, CRISPR-based methods are already being explored to act on pathways that promote fibrosis, focusing not only on controlling symptoms but also on personalized therapies^[6]^. When combined, gene silencing and natural compounds may not only target early disease signals but also limit long-term tissue scarring. Together, these interventions provide hope for expanding treatment choices, especially for patients who currently have very limited options.

Although positive results have been observed, several barriers still limit the practical use of Cas13-based therapies in renal fibrosis. A key issue is achieving efficient, cell-specific delivery to kidney tissues, as current viral and nanoparticle systems raise biological and safety concerns^[6]^. The risk of collateral RNA cleavage and unintended transcript targeting may also disrupt normal cellular functions. Immune and toxic responses have also been reported in preclinical studies, showing the need for careful testing before patient use^[5]^. On top of this, differences in absorption and metabolism of polyphenols make it difficult to ensure consistent results. Overcoming these challenges will require optimized delivery platforms, safer Cas variants, and strong translational studies before such strategies can be applied in clinical practice.

Unlike conventional treatments that mostly slow disease, this approach works on the actual fibrotic drivers while also giving kidney protection^[1,2]^. Still, there are hurdles. First, delivering it directly to kidney cells is difficult; so, research on safer and more targeted carriers should be supported^[2]^. Second, most results are from laboratory models, not patients; this highlights the importance of conducting larger clinical trials^[4,5]^. Third, the long-term safety of gene editing and polyphenol use is still unclear; regulators should ensure close safety checks as studies move forward^[1,6]^.

In conclusion, combining RNA gene silencing with natural protective agents could be a novel strategy against renal fibrosis, shifting nephrology from symptom control to addressing root causes. Despite remaining challenges, current evidence points to a hopeful future for gene-editing tools with bioactive compounds, offering new treatment options for patients with CKD who have limited choices.

## Data Availability

Not applicable to this study.
